# Controlling the Collective Behaviors of Ultrasound-Driven Nanomotors via Frequency Regulation

**DOI:** 10.3390/mi15020262

**Published:** 2024-02-10

**Authors:** Zhihong Zhao, Jie Chen, Gaocheng Zhan, Shuhao Gu, Jiawei Cong, Min Liu, Yiman Liu

**Affiliations:** 1 Hubei Engineering Research Center of Weak Magnetic-Field Detection, College of Science, China Three Gorges University, Yichang 443002, China; 202107020021025@ctgu.edu.cn (Z.Z.);; 2 School of Mechanical Engineering, Jiangsu University, Zhenjiang 212013, China; jw.cong11@gmail.com

**Keywords:** nanomotors, collective behavior, frequency regulation, assembly

## Abstract

Controlling the collective behavior of micro/nanomotors with ultrasound may enable new functionality in robotics, medicine, and other engineering disciplines. Currently, various collective behaviors of nanomotors, such as assembly, reconfiguration, and disassembly, have been explored by using acoustic fields with a fixed frequency, while regulating their collective behaviors by varying the ultrasound frequency still remains challenging. In this work, we designed an ultrasound manipulation methodology that allows nanomotors to exhibit different collective behaviors by regulating the applied ultrasound frequency. The experimental results and FEM simulations demonstrate that the secondary ultrasonic waves produced from the edge of the sample cell lead to the formation of complex acoustic pressure fields and microfluidic patterns, which causes these collective behaviors. This work has important implications for the design of artificial actuated nanomotors and optimize their performances.

## 1. Introduction

Micro/nanomotors, tiny devices converting external energy into motion and force, have gained attention for applications in micro/nanofabrication, environmental remediation, and biomedicine [1,2,3,4,5,6,7,8,9,10,11]. Collective behavior, a common occurrence in nature, involves cooperative actions such as aggregation and transportation in ant colonies, as well as collective defense and offensive behaviors in African hyenas [12]. Inspired by these natural phenomena, researchers have developed micro/nanomotors with diverse driving forces to collaboratively tackle complex tasks. Over the years, various energy sources, including magnetic force [13,14,15,16,17,18,19], gradient energy [1,3,20,21,22,23,24], and ultrasonic energy [25,26,27,28,29,30,31,32,33], have been employed to power these motors. Ultrasonic energy, in particular, is promising due to its non-invasive nature, causing no harm to organisms, and its ability to operate without physical contact. Thus, acoustic-based micro/nanomotors have been widely applied in the field of biomedicine. For example, Yong Luo and others introduced a portable acoustic-driven platform with functionalized microspheres for effective biomarker enrichment and fluorescence enhancement [34]. Integrating capillary technology with ultrasound to induce biomarker enrichment in nano-liter samples [35]. And, combining an integrated circuit system for ultrasound output with a cell-phone-based surface-enhanced Raman scattering (SERS) system enables direct hand-held detection of COVID-19 nucleic acid in nanoliter samples without PCR [36].

In current research, scientists actively manipulate the collective behavior of micro/nanomotors by regulating transducer activation or controlling the intensity of ultrasonic energy [28]. As summarized in Table 1, the research group led by Wang Wei discovered that under the control of a resonant frequency of 4 MHz acoustic field, rod-shaped micromotors with asymmetric structures can exhibit different motion behaviors, such as chain assembly and axial rotation, directional motion and in-plane rotation [25]. Suzanne Ahmed discovered that by controlling the synthesis of rod-shaped nanomotors to possess shape or density asymmetry, it is possible to utilize an acoustic field to drive the directional motion of nanomotors [27]. Tailin Xu’s research indicates that by constructing a standing wave acoustic field in the vertical direction, nanomotors migrate toward the low-pressure region due to the pressure gradient generated acoustically [28]. Qiang Gao has developed a spherical micromotor that can exhibit self-rotation or orbit around a fixed axis when driven by an acoustic field [31]. Xiao Long Lu’s group influences the aggregation or dispersion of micro/nanomotor collectives through the precise control of transducer activation [29]. Similarly, Ze Sheng Li’s group observed micro/nanomotors exhibiting dynamic self-organization, forming intricate flower-like clusters when exposed to a 720 kHz acoustic frequency [30]. Although specific ultrasound frequencies have been applied to drive various micro/nanomotors [31,33], there has been limited research on controlling their collective behavior by controlling ultrasound frequencies. Regulating ultrasound frequency holds the potential to introduce a wider range of patterns in micro/nanomotor collective behavior and create continuous variations in motion. As of now, effectively controlling the collective behavior of micro/nanomotors using ultrasound frequency adjustments remains unresolved.

In this study, we conducted experiments in which nanomotors demonstrated distinct collective behaviors through the continuous modulation of ultrasound frequencies. The nanomotors composed of GaOOH (Gallium oxide hydroxide) with a rod-shaped morphology are capable of autonomous assembly into different patterns, reconfiguration, and disassembly by continuously changing the frequency of the applied ultrasonic field. The collective behaviors include clustering together, moving in stripe-like formations, and vortex-shaped rotation. The experimental results and FEM simulations demonstrate that the secondary ultrasonic waves at the interface between solids and liquids lead to the formation of complex acoustic pressure fields and microfluidic patterns, thereby causing these collective behaviors. This work offers valuable insights that could pave the way for the development of next-generation ultrasonically controlled micromotors and their integration into various applications.

## 2. Materials and Methods

Gallium was purchased from Huatai Metal Materials Technology Co., Ltd. (Dongguan, China), other chemicals used in experiments were from Zechuan Technology Co., Ltd. (Hubei, China), and deionized water was obtained from YA·ZD·5 water distiller from Baolan Experimental Instrument Manufacturing Co., Ltd (Shanghai, China)**.** Rod-shaped GaOOH (Gallium oxide hydroxide) nanomotors were synthesized using an acoustic-assisted physical dispersion method. Initially, 5 g of gallium metal was added to 50 mL of deionized water. Subsequently, the solution was treated with ultrasonic waves (400 W) at 40 degrees Celsius for 30 min. After that, the suspension was centrifuged at 3000 rpm for 15 min. The resulting GaOOH (Gallium oxide hydroxide) nanomotors were washed three times with water and twice with alcohol. The piezoelectric transducer which produces the ultrasound waves (PZT5 disc, 20 mm in diameter, and 0.5 mm in thickness, Shenlei Ultrasonic Equipment Co., Ltd. (Shaoxing, China)) was attached to the bottom center of a glass slide. The continuous ultrasound sine wave was applied via a piezoelectric transducer, through a Tektronix AFG3102C (Tektronix Technology (China) Co., Ltd. (Shanghai, China)) dual-channel arbitrary waveform generator, in connection to an FPA301 power amplifier (Feiyi Technology Co., Ltd. (Zhengzhou, China)). All experiments were observed in a two-dimensional plane near the bottom surface and recorded using an inverted optical microscope (Zeiss Axio BA600 microscope with 10×, 20×, 50×, and 100× objectives, Carl Zeiss Optics (China) Co., Ltd. (Guangzhou, China)) equipped with a Photometrics ModelGP-550H CCD camera (Yingshang Xingyuan Technology Development Co., Ltd. (Fuyang, China)). The electrical signal was monitored using a 60 MHz Tektronix TDS1002 (Tech Technology (China) Co., Ltd.(Shanghai, China)) storage oscilloscope.

Characterization: Scanning electron microscope (JSM-7500F SEM, Nippon Electronics Co., Ltd. (Beijing, China)) and transmission electron microscope (JEOL F200 TEM, Nippon Electronics Co., Ltd. (Beijing, China)) with energy dispersive spectroscopy system were employed to characterize the morphology and structure information of nanomotors. The near-infrared absorption (NIR) spectrum was recorded using a UV-3600 iPlus UV visible near-infrared spectrophotometer (Shimadzu Enterprise Management (China) Co., Ltd. (Shanghai, China)). X-ray photoelectron spectroscopy (XPS) was analyzed using an AXIS Supra X-ray photoelectron spectroscopy (Shimadzu Enterprise Management (China) Co., Ltd. (Shanghai, China)). The X-ray diffraction (XRD) patterns of the samples were recorded using a SmartLab (9k) X-ray diffractometer (Rigaku Corporation (Tokyo, Japan)). The Microtrac S3500 laser particle size analyzer (Microtrac MRB (Montgomeryville, PA, USA)) was used to conduct particle size distribution analysis on the experimental samples.

## 3. Results and Discussion

**Figure 1:**
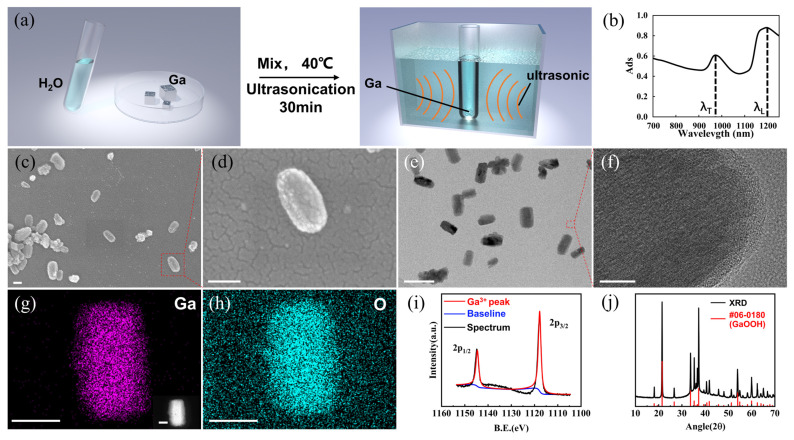
Preparation and characterization of rod-shaped nanomotors. (**a**) Scheme of the fabrication of Ga-based nanomotors. (**b**) Absorption spectrum of the prepared nanomotors. (**c**,**d**) SEM image (scale bar: 100 nm) and (**e**) TEM image of nanomotors (scale bar: 200 nm). (**f**) High-resolution TEM image of a single nanomotor (scale bar: 10 nm). (**g**,**h**) EDX mapping images showing the distribution of Ga and O elements in a single nanomotor (scale bar: 100 nm). Inset: the corresponding SEM image (scale bar: 100 nm). (**i**) XPS spectrum and (**j**) XRD pattern of the nanomotors.

The shape and material play a critical role in the manipulation of nanomotors [25]. Due to the structural asymmetry of rod-shaped nanomotors, they exhibit greater sensitivity to ultrasound compared to other shapes of micro/nanomotors. Additionally, gallium has excellent biocompatibility and is easy to process. Therefore, we chose to synthesize rod-shaped GaOOH (Gallium oxide hydroxide) as samples. The nanomotors were fabricated using ultrasonic treating Gallium at 40 °C for 30 min, as schematically illustrated in Figure 1a. Figure 1b presents the near-infrared absorption (NIR) spectrum of the resulting nanomotors. The NIR spectrum reveals that the transverse peak (λ_T_) of nanomotors is found at 980 nm, while the longitudinal NIR peak (λ_L_) is at 1200 nm. Scanning electron microscopy (SEM) and transmission electron microscopy (TEM) (Figure 1c–f) are employed to image and evaluate the nanostructures of the nanomotors. Figure 1c–e illustrate that most nanomotors exhibit a rod-shaped structure, with only a small portion appearing as irregular shapes. These rod-shaped nanomotors, with some small shape asymmetries, have been determined to have a length of 150 ± 10 nm and a diameter of 100 ± 10 nm. To examine the uniformity of the prepared nanomotors in size, we conducted particle size distribution analysis, and the results are shown in Figure S2. The size distribution of the prepared nanomotors mainly falls within the range of 100 nm to 200 nm, comprising 90% of the total proportion. Among them, particles around 150 nm diameter exhibit the highest proportion (>40%). It is evident that the prepared nanomotors using the above-mentioned method exhibit relatively good consistency in size. In addition, the high-magnification SEM image in Figure 1d provides a detailed view of a single rod-shaped nanomotor. The surface structure of a rod-shaped nanomotor is characterized by High-Resolution Transmission Electron Microscope (HRTEM) in Figure 1f, displaying lattice fringes that confirm the crystal nature of these GaOOH (Gallium oxide hydroxide) nanomotors (Figure S1). Energy Dispersive Spectroscopy (EDS) was employed for the analysis of the nanomotors’ composition. As depicted in EDS mapping (Figure 1g,h), gallium and oxygen elements are evenly distributed on the nanorod. To further investigate the valence states of gallium elements in nanomotors, X-ray photoelectron spectroscopy (XPS) analysis was carried out. The XPS survey scans (Figure 1i) show that all gallium in the nanomotor exhibits trivalent behavior, indicating complete oxidation of gallium elements in the nanomotors. X-ray diffraction (XRD) studies were performed to identify the composition and purity of the nanomotors, revealing that the chemical composition is GaOOH (Gallium oxide hydroxide), a typical form of gallium oxide formed when gallium undergoes oxidation in water (Figure 1j).

**Figure 2:**
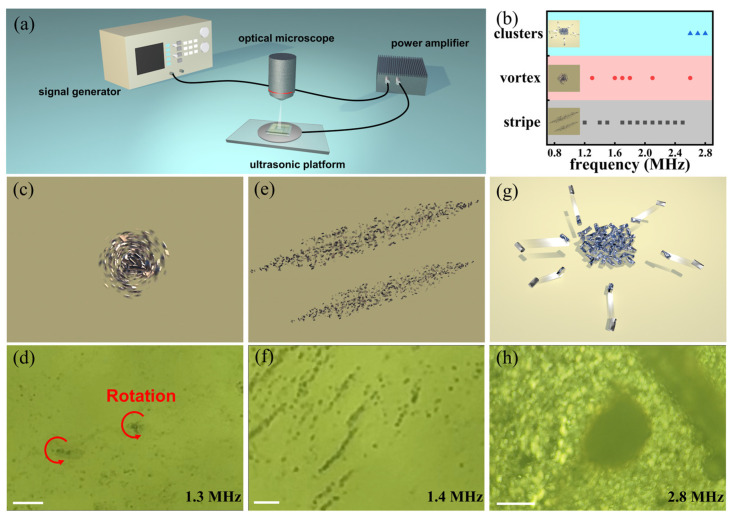
The collective behaviors of the GaOOH (Gallium oxide hydroxide) nanometers under the ultrasonic field using different frequencies. (**a**) Scheme of the experimental setup. The ultrasonic platform consists of a signal generator, a power amplifier, the ultrasonic platform, and a microscope for observation. (**b**) The collective behavior of nanomotors varies with the ultrasound frequency. Schematic diagrams (**c**,**e**,**g**) and experimental results (**d**,**f**,**h**) of three patterns of collective behavior. (**d**) Nanomotors exhibit a vortex-shaped pattern at an ultrasound frequency of 1.3 MHz; Scale bar: 20 µm. (**f**) Nanomotors exhibit stripe-like chains at a frequency of 1.4 MHz; Scale bar: 10 µm. (**h**) Nanomotors exhibit a cluster pattern at an ultrasound frequency of 2.8 MHz; Scale bar: 100 µm.

We tested the collective performance of these nanomotors under different input ultrasound frequencies. Experiments were conducted using a homemade ultrasonic manipulation platform, as illustrated in Figure 2a. It integrates a signal generator for customizable frequency signals, a power amplifier, and a homemade ultrasonic platform (cell width: 5.2 mm, length: 5.8 mm, height: 260 μm). Videos were taken using an optical microscope (ocular lenses: 10×, objective lenses: 20×~100×). The observed collective behaviors can be broadly categorized into three distinct patterns: stripe-like chains, vortex-shaped patterns, and relatively stable clusters. As seen in Figure 2c,d, nanomotors gathered into small clusters and formed a vortex-shaped pattern at a 1.3 MHz ultrasonic field frequency (Video S1, Part 1) with very fast in-plane rotations. While at a driving frequency of 1.4 MHz, these nanomotors form parallel stripes with fast rotations along the axis of the stripes. Representative observations of this stripe-like pattern are shown in Figure 2e,f (taken from Video S1, Part 2). Interestingly, after tuning the frequency away from this frequency range (for example, frequency = 2.8 MHz), these nanomotors aggregated progressively (Figure 2g,h, Video S1, Part 3). Turning off the acoustic field caused the nanomotors to redisperse into a suspension uniformly (Video S1, Part 3). Obviously, varying the frequency could lead to the transformation between these collective patterns. As demonstrated in Video S2, tuning the frequency from 2.4 MHz to 2.6 MHz shifted the nanomotors’ collective behavior from stripe-like chains to vortex-shaped rotation. When the frequency changes from 2.6 MHz to 2.8 MHz, the nanomotors aggregated progressively. The transformation behaviors are summarized briefly in Figure 2b. As seen, the nanomotors exhibited alternating motion between stripe-like chains and vortex-shaped patterns in the 1 MHz–2.6 MHz range, transforming into clusters in the frequency range from 2.6 MHz to 2.8 MHz.

**Figure 3:**
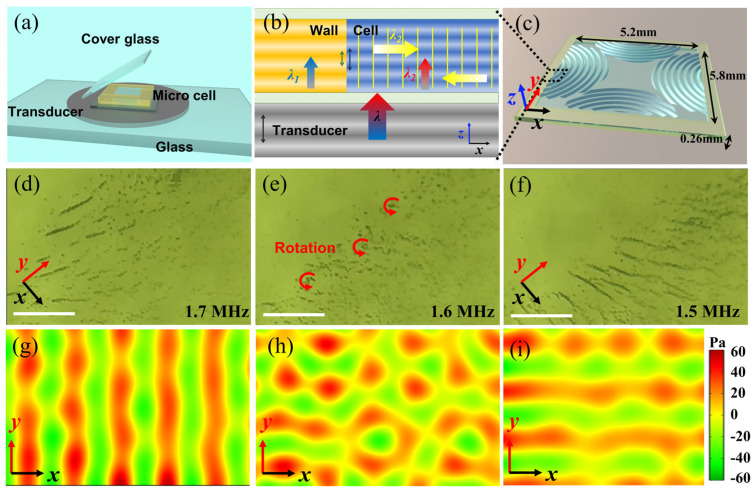
Propagation characteristics of ultrasonic waves and acoustic pressure field distribution within the ultrasonic manipulation platform at different frequencies. (**a**) The ultrasonic manipulation platform is composed of a substrate glass, transducer, microfluidic cell, and cover glass. (**b**) The ultrasonic waves in the vertical direction generate secondary waves in the horizontal direction at the solid–liquid interface. (**c**) Schematic diagram of a microfluidic cell with dimensions of 5.2 mm × 5.8 mm × 0.26 mm. (**d**–**f**) The corresponding collective behaviors at ultrasound frequencies of 1.7 MHz, 1.6 MHz, and 1.5 MHz; Scale bar: 50 µm; Corresponding to Video S3. (**g**–**i**) Simulation diagrams of the acoustic pressure field in the horizontal plane at frequencies of 1.7 MHz, 1.6 MHz, and 1.5 MHz.

The collective behaviors of these nanomotors are highly influenced by the geometric parameters of the sample cell and the applied ultrasound frequency. In our experiment, the ultrasonic platform consists of several layers: the bottom transducer, covered by a glass slide for a smooth surface; a micro-water cell (5.8 mm length, 5.2 mm width, 260 μm height) enclosed with tape (Biaxially oriented polypropylene film, BOPP); and a top glass slide to reflect ultrasonic waves (Figure 3a). The bottom transducer generates continuous ultrasonic waves along the *z*-axis in isotropic elastic media (for example, in water and tape). While at the edge of the cell, interaction-induced secondary waves arise due to the non-uniformity of the medium, as depicted in Figure 3b. The different densities of the liquid and the wall (composed of adhesive tape) cause different velocities and wavelengths. As a consequence, oscillation coupling between the wall and water generates ultrasonic waves along the *x*-axis and *y*-axis using the same frequency. The in-plane waves continuously reflect within the *x-o-y* plane of the cell (Figure 3c). It is well-known that the stripe-like chains’ pattern, regardless of their direction, is usually considered to be the result of collecting nanomotors at the nodes of ultrasonic standing waves, and the motion of the nanomotors is driven by the acoustic pressure fields [25]. Thus, changing the frequency alters the position of in-plane wave nodes, which induces different collective behaviors of the nanomotors. 

To illustrate the critical role of ultrasound frequency in achieving different patterns, we further carefully compared the collective behaviors of nanomotors in an ultrasonic field using frequencies from 1.5 to 1.7 MHz. When the frequency is set at 1.7 MHz (Figure 3d, corresponding to Video S3), standing ultrasound waves exclusively exist along the *x*-axis, with their trajectory parallel to the *y-o-z* plane. Consequently, nanomotors are observed displaying stripe-like patterns similar to the *y*-axis while simultaneously spinning around their own axis, which is generally considered to be due to the interference of traveling waves along the *z*-axis. However, a slight deviation away from the resonant frequency (1.6 MHz) dramatically decreased the intensity of the standing waves in the cell, and thus only traveling waves can be observed in the *x-o-y* plane. In this state, the microfluidic field generated by the disturbance of these traveling waves induces nanomotors to exhibit a vortex-shaped collective behavior (Figure 3e). As the input frequency decreases to 1.5 MHz, the standing waves form along the *y*-axis. At certain frequencies, the nanomotors formed the stripe-like aggregates that parallel the *x*-axis within seconds, as shown in Figure 3f and the corresponding Video S3. Such transformation indicates that the aggregation of these nanomotors into a defined pattern can be attributed to the distribution of acoustic pressure fields in the cell.

Based on the theoretical model of ultrasound [32], the conditions for forming standing waves in an enclosed space can be represented by

(1) $$s_{i}=\frac{1}{2}n\lambda_{i}=\frac{1}{2}n\frac{c}{f_{i}};\;n=1,2,3\ldots ;i=x,y,z$$

Here, *s* represents the characteristic length of ultrasonic wave transmission, and *c* denotes the speed of sound in the medium. In our experiment, *c* corresponds to the speed of sound in deionized water, specifically c = 1492 m/s, and thus the conditions for the formation of standing waves for in-plane and out-of-plane waves can be calculated using Equation (1) as follows:

(2) $$f_{x}=0.143n\;MHz\;;\;n=1,2,3\ldots$$

(3) $$\;f_{y}=0.128n\;MHz\;;\;n=1,2,3\ldots$$

(4) $$f_{z}=2.86n\;MHz;\;n=1,2,3\ldots$$

The ultrasonic waves continuously reflected and superimposed in the square cell, leading to the formation of complex acoustic pressure fields and microfluidic patterns, thereby resulting in the diverse collective behavior of nanomotors.

To further investigate this mechanism, we simulated the acoustic pressure field using a multi-physics field simulation approach based on the finite element method. The acoustic equation (Helmholtz equation), as described by Louisnard [37], which ignores thermal and viscous losses due to cavitation, is expressed as follows:

(5) $$\nabla \cdot \left(\frac{1}{\rho }\nabla p\right)+k^{2}p=0$$

Here, p represents the acoustic pressure, ρ is the density of the liquid, and k is the wave number. In solid boundaries, the solid mechanics equation describes how an object deforms and moves under the influence of forces. Specifically, the linear elasticity equation is expressed as follows:

(6) $$\rho_{s}\frac{\partial^{2}u}{\partial t^{2}}-\nabla \cdot \sigma =0$$

*u* represents the displacement field, describing the displacement of points within the object, *$\rho_{s}$* is the density of the solid, and *σ* is the stress tensor, indicating the internal forces at different points within the object. And acoustic solid–liquid coupling term is as follows:

(7) −∇p·n=σ·n

This term represents the interaction between the acoustic field and the solid object, where *n* is the outward normal vector. The two-dimensional distribution maps of the acoustic pressure field are obtained by applying the finite element method to solve the aforementioned equations. It is noteworthy that, besides ultrasound frequency, input voltage also significantly influences the behavior of nanomotors. Extensive research has shown that the motion patterns of nanomotors are influenced by both the input ultrasound frequency and the structure of the nanomotors, while the input voltage primarily affects the movement rate of nanomotors [25,26,27]. To determine the most suitable experimental voltage, we varied the input voltage at a fixed frequency to observe the motion of nanomotor clusters. On our ultrasound manipulation platform, observing nanomotor cluster motion is challenging when the voltage is below 6 V. However, when the voltage exceeds 6 V, the rotation rate of nanomotors gradually increases with the increasing input voltage. To achieve optimal experimental results, 12 V was selected as the input voltage for all experiments and FEM simulations.

Figure 3g illustrates the two-dimensional acoustic pressure field distribution within the *x-o-y* plane of cells at a resonant frequency of 1.7 MHz, in which the standing wave planes parallel to the *y-o-z* plane. This is consistent with our observation (Figure 3d). Figure 3h displays the acoustic pressure field with a frequency of 1.6 MHz, in which standing waves are not observed. At that frequency, nanomotors were trapped by traveling waves along the *y*-axis and *x*-axis simultaneously, and thus autonomously assembling into many small vortices that rotate together in a synchronized manner, as illustrated in Figure 3e. Figure 3i depicts the acoustic pressure field with a frequency of 1.5 MHz, similar to that in 1.7 MHz, except for the direction of the stripes.

It is crucial to emphasize that these simulated results were limited to the *x-o-y* plane. Because the ultrasound manipulation platform used here has a very small vertical scale (260 μm), the minimum frequency for standing waves in *z*-direction waves is significantly higher than the minimum frequency for in-plane (*x-o-y* plane) waves (~2.86 MHz). When the ultrasound frequency becomes close to 2.86 MHz, the out-of-plane waves propagate along the *z*-axis and are reflected back from the cover glass giving rise to a standing wave. At this state, the nanomotors levitated into the nodal plane, forming small clusters. The origin of the levitation force is generally attributed to the primary acoustic radiation force exerted on the nanomotors by sound propagation perpendicular to the substrate [25]. A slight deviation away from 2.86 MHz caused the nanomotors to redisperse into a suspension, as illustrated in Supporting Video S1, Part 3.

**Figure 4:**
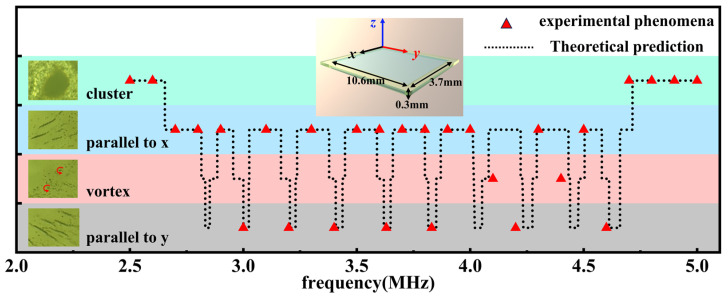
Predicted and observed collective behavior patterns of nanomotors at different frequencies. The predicted collective behavior of nanomotors at different ultrasound frequencies is represented by black dashed lines, while the experimentally observed phenomena are denoted by red dots. Inset: Schematic diagram of a cell with dimensions 3.7 mm × 10.6 mm × 0.3 mm.

As we mentioned above, this mechanism discussed above allows us to change the collective behaviors of the nanomotors by independently adjusting the geometric parameters of the sample cells. To further investigate this mechanism, we fabricated another sample cell with dimensions of 3.7 mm width in the *x*-direction, 10.6 mm length in the *y*-direction, and 0.3 mm height in the *z*-direction. It is easy to predict the collective behaviors of nanomotors in such a cell. Figure 4 shows the theoretical prediction and experimental results. As expected, the experimental results show a high degree of conformity with the predicted phenomena. When the frequency is adjusted to approximately 2.48 MHz or 4.96 MHz, standing waves manifest in the vertical direction, giving rise to observed clustering phenomena. In addition, both stripe-like and vortex-shaped patterns were effectively observed by tuning the ultrasound frequency in the range from 2.48 MHz to 4.96 MHz. Because the configuration of our transduce and cell are not yet optimized, it seems reasonable to conclude that more collective patterns will be possible in different sample cells with more complex geometries.

## 4. Conclusions

In conclusion, we have presented a general approach to control the collective behavior of ultrasound-driven nanomotors by tuning the ultrasound frequency. The GaOOH (Gallium oxide hydroxide) nanomotors are capable of autonomous assembly, reconfiguration, and disassembly in response to changes in the applied frequency of the ultrasound field in a square sample cell. It was demonstrated that the secondary ultrasonic waves produced from the edge of the sample cell led to the formation of complex acoustic pressure fields and microfluidic patterns that cause these collective behaviors. In contrast to commonly used methods that involve adjusting the intensity of ultrasound with a fixed frequency, the reported method demonstrates greater flexibility, offering different collective behavior patterns and enabling precise control of nanomotor movement across a broader range of frequencies. It not only offers fresh perspectives for the innovation of ultrasound manipulation platforms but also has the potential to inspire the development of diverse ultrasound-driven micro/nanomotors for accomplishing more intricate tasks.

## Figures and Tables

**Table 1:** Various motion behaviors based on ultrasound driving.

Motion Behaviors	Morphology	Reference
Chain assembly and axial rotation, directional motion, and in-plane rotation	Microrods	[25]
Directional motion	Microrods	[27]
Gathering and transfer	Nanorods	[28]
Disperse and aggregate	Microrods	[29]
Self-organize	Microrods	[30]
Self-rotation around a fixed axis	Spheric Janus	[31]
In-plane rotation and spinning	Microrods	[33]

## Data Availability

Data are contained within the article and supplementary materials.

## Supplementary Materials

The following supporting information can be downloaded at: https://www.mdpi.com/article/10.3390/mi15020262/s1, Video S1: Three distinct collective behaviors of nanomotors in ultrasonic field with different frequencies. Video S2: The transformation of collective behavior between different patterns. Video S3: Changing the direction of stripe-like patterns from the *x*-axis direction to the *y*-axis direction. Figure S1: Lattice diffraction patterns captured by high-resolution transmission electron micros-copy. Figure S2: Lattice diffraction patterns captured by high-resolution transmission electron microscopy.
