# Supplementary material for: Controlling the Collective Behaviors of Ultrasound-Driven Nanomotors via Frequency Regulation
Source: Micromachines (Basel). 2024 Feb 10;15(2):262. doi: 10.3390/mi15020262 (PMC10892131; doi:10.3390/mi15020262)
Supplement: Supplementary file 1 [file micromachines-15-00262-s001.zip › Supporting information.pdf]

Controlling the Collective Behaviors of Ultrasound-Driven Nanomotors by Frequency Regulation

Zhihong Zhao [1], Jie Chen [1], Gaocheng Zhan [1], Shuhao Gu [1], Jiawei Cong [2], Min Liu [1,*] and Yiman Liu [1,*]

[1] Hubei Engineering Research Center of Weak Magnetic-Field Detection, College of Science, China Three Gorges University, Yichang 443002, China
[2] School of Mechanical Engineering, Jiangsu University, Zhenjiang 212013, China
* Correspondence: lmin@ctgu.edu.cn (M.L.); lym@ctgu.edu.cn (Y.L.)

Supporting Videos

Video S1. Three distinct collective behaviors of nanomotors in ultrasonic field with different frequencies. Part 1, Vortex-shaped rotation (1.3MHz); Part 2, Stripe-like chains (1.4 MHz); Part 3, Assembly into clusters and disassembling (2.8MHz).

Video S2. The transformation of collective behavior between different patterns.

Video S3. Changing the direction of stripe-like patterns from the *x*-axis direction to the *y*-axis direction.

Supporting Figures

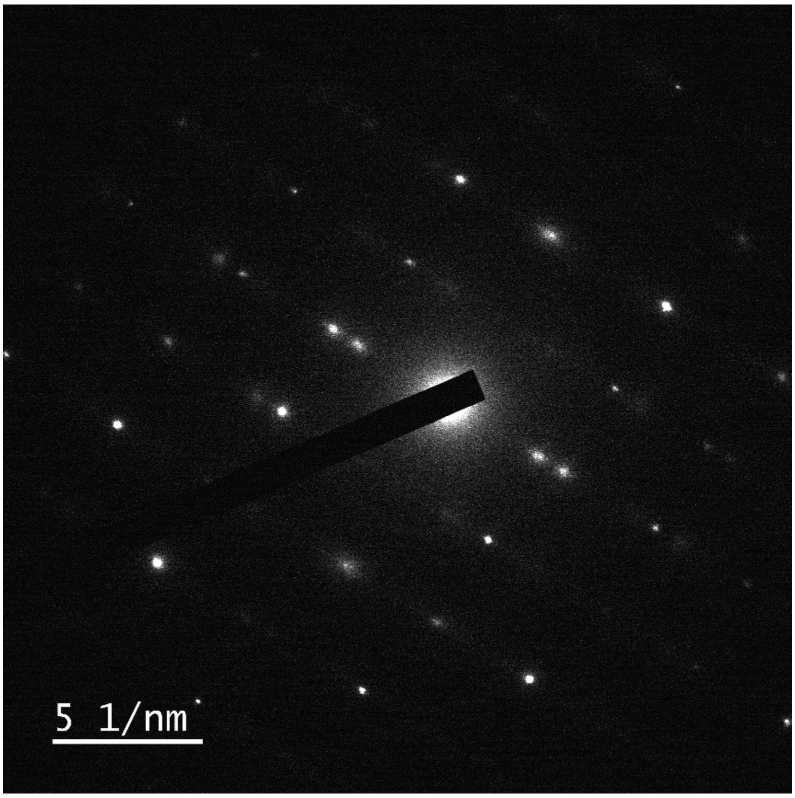

Figure S1. Lattice diffraction patterns captured by high-resolution transmission electron microscopy.

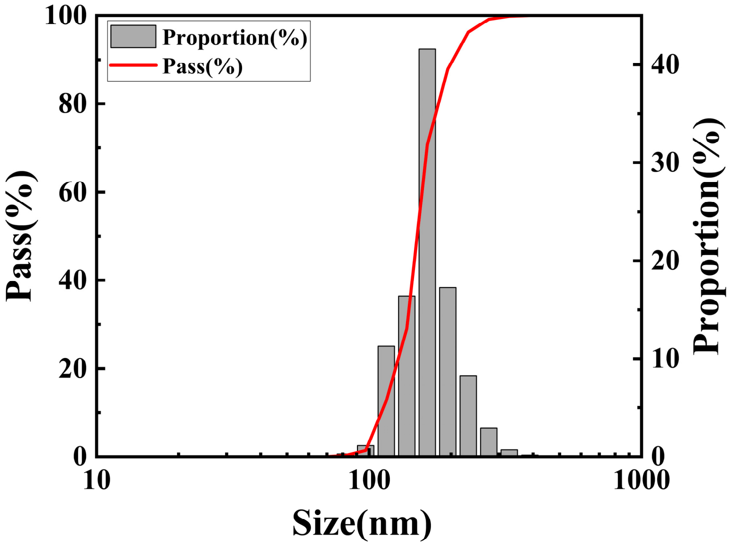

Figure S2. Lattice diffraction patterns captured by high-resolution transmission electron microscopy
